# A Machine That Measures Thought

**Published:** 1882-05

**Authors:** 


### A MACHINE THAT MEASURES THOUGHT.

A machine has been invented by Doctor Mosso, of Turin, which measures thought. It is called the plethysmograph, and its revelations are based on the fact that thought creates nervous action, which consumes in its performance a certain quantity of blood, and that quantity may be measured. In an address before the American Association of Science, Professor G. F. Barker describes the machine and its working as follows:

The forearm, for example, being the organ to be experimented on, is placed in a cylinder of water and tightly inclosed. A rubber tube connects the interior of the cylinder with the recording apparatus. With the electric circuit by which the stimulus was applied to produce contraction were two keys, one of which was a dummy. It was noticed that, after using the active key several times, producing varying current strengths, the curve sank as before on pressing down the inactive key. Since no real effect was produced, the result was caused solely by the imagination, blood passing from the body to the brain in the act.

To test further the effect of mental action, Doctor Pagliani, whose arm was in the apparatus, was requested to multiply two hundred and sixty-seven by eight mentally, and to make a sign when he had finished. The recorded curve showed very distinctly how much more blood the brain took to perform the operation. Hence the plethysmograph is capable of measuring the relative amount of mental power required by different persons to work out the same mental problem. Indeed, Mr. Gaskel suggests the use of this instrument in the examination room, to find out, in addition to the amount of knowledge a man possesses, how much effort it causes him to produce any particular result of brain-work.

Doctor Mosso relates that, while the apparatus was set up in his room in Turin, a classical man came in to see him. He looked very contemptuously upon it, and asked of what use it could be, saying that it couldn't do anybody any good.

Doctor Mosso replied: "Well, now I can tell you by that whether you read Greek as easily as you can Latin."

As the classicist would not believe it, his own arm was put into the apparatus, and he was given a Latin book to read. A very slight sinking of the curve was the result.

The Latin book was then taken away, and a Greek book was given to him. This produced immediately a much deeper curve.

He had asserted before that it was quite as easy for him to read Greek as Latin, and that there was no difficulty in doing either. Doctor Mosso, however, was able to show him that he was laboring under a delusion.

Again, this apparatus is so sensitive as to be useful for ascertaining how much a person is dreaming. When Doctor Pagliani went to sleep in the apparatus, the effect upon the resulting curve was very marked indeed. He said afterward that he had been in a sound sleep, and remembered nothing of what had passed in the room—that he had been absolutely unconcious; and yet, every little movement in the room, such as the slamming of a door, the barking of a dog, and even the knocking down of a piece of glass, were all marked on the curves. Sometimes he moved his lips, and gave other evidences that he was dreaming. They were all recorded on the curve, the amount of blood required for dreaming diminishing that in the extremities.

---

FRIENDSHIP is a good deal like china. It is very durable and beautiful as long as it is quite whole; but break it and all the cement in the world will not quite repair the damages. You may stick the pieces together, so that at a distance it looks as well as ever; but it won't hold hot water. It is always ready to deceive you, if you trust it; and it is on the whole a very worthless thing, fit only to be set empty upon a shelf and forgotten there. The finer and more delicate it is, the more utter the ruin. A mere acquaintance, which needs only a little ill-humor to break it up, may be coarsely puttied, like that old yellow basin in the store closet, but tenderness and trust and sweet exchange of confidence can no more be yours when angry words and thoughts have broken them, than can those delicate porcelain tea-cups which were splintered to pieces be restored to their original handsome excellence. The slightest crack will spoil the true ring, and you had better search for a new friend than try to mend the old one. And all this has nothing to do with forgiveness. One may forgive and be forgiven; but the deed has been done and the word said. The flowers and gilding are gone. The formal "making-up," especially between two women, is of no more avail than the wonderful cements that have made a cracked ugliness of the china vase that you expected to be your "joy forever." Handled delicately, washed to purity in the waters of truth, confided to no careless, unsympathizing hands, friendship may last two lives out, but "it does not pay" to try to mend it. Once broken, it is spoiled forever.

### DISGUISED CABBAGENIANS.

SAYS the Boston *Transcript*: "They used to say that a good cigar could be known by the light brown specks on it. These were made by worms, the story was, that the worms were epicures in tobacco and would touch only the best; but the chemists soon found a way of simulating these worm specks. So that spoiled that test. Then there was no other guide but the ashes. If they burned white the cigar was good; if not, bad. But the enterprising tobacconist soon found a way to make the vilest cabbagenia burn as spotlessly white as the best Havana. Another test gone. Finally the makers of choice cigars put a little red label around each. This was thought to be something which would always be a sure guide, and so it would be, but unfortunately some of the manufacturers have, by a strange mistake, put the labels on the cabbagenias as well as the Havanas.

CATARRH.—A health publication says one of the most prominent causes of catarrh is the excessive use of salt and heaters, such as sweets, fats, condiments and starch. This excess of carbonaceous food excites and inflames the mucous membranes of the throat, nasal passages, bronchial tubes, etc. It is also caused by indigestion and constipation. Headaches and neuralgia often proceed from the same causes.

